# Analysis of telomere length and function in radiosensitive mouse and human cells in response to DNA-PKcs inhibition

**DOI:** 10.1186/2041-9414-4-2

**Published:** 2013-03-22

**Authors:** Hemad Yasaei, Yaghoub Gozaly-Chianea, Predrag Slijepcevic

**Affiliations:** 1 Division of Biosciences, Brunel Institute of Cancer Genetics and Pharmacogenomics, School of Health Sciences and Social Care, Brunel University, Uxbridge, Middlesex, UB8 3PH, UK

**Keywords:** Telomere length, DNA-PKcs, Artemis, Mouse lymphoma, Telomere dysfunction, IC86621, Flow-FISH, Radiosensitivity

## Abstract

**Background:**

Telomeres, the physical ends of chromosomes, play an important role in preserving genomic integrity. This protection is supported by telomere binding proteins collectively known as the shelterin complex. The shelterin complex protects chromosome ends by suppressing DNA damage response and acting as a regulator of telomere length maintenance by telomerase, an enzyme that elongates telomeres. Telomere dysfunction manifests in different forms including chromosomal end-to-end fusion, telomere shortening and p53-dependent apoptosis and/or senescence. An important shelterin-associated protein with critical role in telomere protection in human and mouse cells is the catalytic subunit of DNA-protein kinase (DNA-PKcs). DNA-PKcs deficiency in mouse cells results in elevated levels of spontaneous telomeric fusion, a marker of telomere dysfunction, but does not cause telomere length shortening. Similarly, inhibition of DNA-PKcs with chemical inhibitor, IC86621, prevents chromosomal end protection through mechanism reminiscent of dominant-negative reduction in DNA-PKcs activity.

**Results:**

We demonstrate here that the IC86621 mediated inhibition of DNA-PKcs in two mouse lymphoma cell lines results not only in elevated frequencies of chromosome end-to-end fusions, but also accelerated telomere shortening in the presence of telomerase. Furthermore, we observed increased levels of spontaneous telomeric fusions in Artemis defective human primary fibroblasts in which DNA-PKcs was inhibited, but no significant changes in telomere length.

**Conclusion:**

These results confirm that DNA-PKcs plays an active role in chromosome end protection in mouse and human cells. Furthermore, it appears that DNA-PKcs is also involved in telomere length regulation, independently of telomerase activity, in mouse lymphoma cells but not in human cells.

## Introduction

Protection of genomic integrity is an essential function required for continued survival of mammalian cells. Telomeres play an important part in maintaining genomic integrity and chromosomal stability. All eukaryotic cells need to distinguish the natural chromosomal ends from exogenously/endogenously induced DNA ends resulting from DNA double strand breaks (DSBs) [1]. DNA DSBs in mammalian cells are processed by two mechanisms - non-homologous end joining (NHEJ) and homologous recombination (HR). It is now well documented that loss of telomeric function leads to chromosomal end-to-end fusions and can trigger a cell cycle arrest and apoptosis [2]. Therefore dysfunctional telomeres are detected as DNA damage by the DNA damage response mechanisms [3,4].

Telomere dysfunction can arise as a result of natural telomere shortening (in the absence of telomerase), or loss of function of the telomere protective protein complex known as shelterin, or loss of function of some DNA damage response proteins [3]. Telomere dysfunction as a result of telomere shortening leads to the physiological process known as replicative senescence, a p53-dependant antiproliferative mechanism. Dividing differentiated human cells lack telomerase, the enzyme that synthesizes telomeric DNA, leading to telomere shortening. When telomeres become critically short in telomerase negative cells, they lose their protective function and are subsequently detected by the DNA damage response proteins as sites of DNA damage. The presence of DNA damage at telomeres precludes further cell division and results in p53-dependent cell cycle arrest. Alternatively, telomere dysfunction can result from pathological mutations in genes encoding (i) components of telomerase, (ii) components of the shelterin complex and (iii) some DNA damage response genes including those involved in NHEJ [5,6].

DNA-PKcs and Ku86 are key components of the NHEJ pathway and have been demonstrated to interact directly with telomeres in mammalian cells. Mice deficient for either of these proteins have increased frequencies of telomere end-to-end fusions without significant loss of telomeric DNA [7,8] indicating that telomere dysfunction can be independent of telomere length. Moreover, double knock-out mice deficient in DNA-PKcs and telomerase show accelerated telomere shortening, suggesting a functional interaction between telomerase and DNA-PKcs in maintenance of telomere length [8,9]. Another NHEJ protein shown to be involved in telomere maintenance is Artemis [10]. Artemis is phosphorylated by DNA-PK complex (DNA-PKcs and Ku70/Ku80) in response to DNA-DSB and the activated endonuclease function of Artemis is thought to be critical in 3’-overhang processing in the NHEJ [11]. A range of DNA damage response proteins involved in other DNA repair, damage signalling and checkpoint pathways have been shown to associate, albeit transiently, with shelterin and telomeres with documented telomere dysfunction phenotypes [5].

The activity of DNA-PKcs can be inhibited using synthetic chemical inhibitors such as 4-(morpholinyl)-4*H*-naphthol[1,2-*b*]pyran-4-one (NU7026) or 1-(2-Hydroxy-4-morpholin-4-yl-phenyl)ethanone (IC86621) [12]. IC86621 has been reported to affect catalytic subunit of DNA-PK with increases in telomere end-to-end fusions [1].

In this study we set out to examine the effect of DNA-PKcs inhibition on telomere maintenance and telomere length regulation in two mouse lymphoma cell lines: the parental radio-resistant L5178Y-R cell line (also known as LY-R) and its subtype, the radiosensitive L5178Y-S cell line (also known as LY-S) [13]. Moreover, we set out to further elucidate the link between DNA-PKcs and Artemis in telomere protection and telomere length regulation in primary human fibroblasts.

## Results and discussion

### Inhibition of DNA-PKcs increases telomeric fusion and dysfunction

LY-R and LY-S cells have been well characterized in terms of DNA damage response capacities and telomere maintenance. LY-R cells show normal sensitivity to ionizing radiation (IR) and have telomeres typical of mouse cells i.e. 40–50 kb [13,14]. In contrast, LY-S cells show IR sensitivity [15] and have much shorter telomeres which are in the region of ~ 7 kb. A recent study indicates that this pair of cell lines may have different telomere lengths in different laboratories [16]. The mechanisms of increased IR sensitivity in LY-S cells are not known. It has been suggested that this radiosensitivity is due to a deficiency in the DSB repair machinery [17]. However, DNA-PKcs and all components of NHEJ pathway known at the time of investigation including Ku70/80 were functional in both cell lines [13,18]. The only components of NHEJ not examined in these cell lines are the more recently discovered proteins XLF/Cernunnos and Artemis.

**Figure 1:**
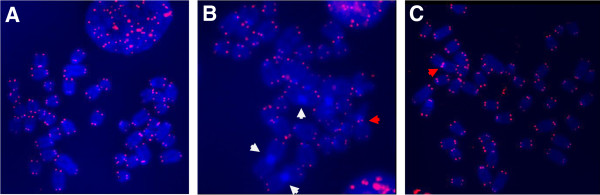
**Examples of chromosomal aberrations in LY-R and LY-S mouse cells. A|** No evidence of chromosomal aberrations in untreated mouse LY-R cells. Telomeric signals are in red with DNA counter stained in blue with DAPI. **B|** Elevated levels of Robertsonian fusion (white arrow) and end-to-end telomeric fusions in DNA-PKcs inhibited mouse LY-S. **C|** Similar observation in DNA-PKcs inhibited treated mouse LY-R. Note the difference in telomeric signal strength between LY-S (weaker signal) and LY-R (stronger) suggesting differences in telomeric length of the two mouse cell lines.

We started by analysing spontaneous chromosome abnormalities in the above cell lines using Telo-FISH. In particular, we were interested in end-to-end chromosome fusions as these can result from telomere dysfunction. Since all mouse chromosomes are acrocentric (Figure 1A) end-to-end chromosome fusions can be of two types: Robertsonian (RB) fusions and classical telomeric fusions. RB fusions are characterized by the fusion involving p-arm telomeres and the lack of telomeric signals at the fusion point as shown in Figure 1B (white arrows) [19]. Classical telomeric fusions are fusions of either p-arm or q-arm telomeres that show clear telomeric signals at fusion points as shown in Figure 1B and C (red arrows) [19]. The mechanisms behind RB fusion and telomeric fusion formation are different. It is likely that RB fusion may arise as a result of telomere shortening, whereas the classical telomeric fusions usually arise as a result of telomere dysfunction not involving changes in telomere length [19].

We observed a significant four-fold increase in frequencies of RB fusions in LY-S cells relative to LY-R cells (p < 0.0001) (Table 1). This increase could be the result of LY-S cells having shorter telomeres (7Kb in LY-S and 49 Kb in LY-R) and higher levels of missing telomeric signals [13].The analysis of telomeric fusions showed a 1.7-fold increase in LY-S cells in comparison to LY-R cells (p < 0.0001). Mouse p-arm telomeres are significantly shorter than q-arm telomeres [19] thus explaining why the frequencies of RB fusions were greater than frequencies of telomeric fusions in untreated LY-S cells (Table 1). However, this does not hold true for LY-R cells (Table 1) suggesting that mechanisms other than telomere shortening may be involved. It is important to note that the presence of critically short telomeres may affect LY-S cells more dramatically than LY-R cells because of their naturally short telomeres.

**Table 1:** Telo-FISH analysis of two mouse cell lines treated with inhibitor of DNA-PKcs

			**Frequency (event/cell)**	
**Cell Line**	***Metaphases Scored***	***RB fusion***	***Telomeric Fusions***	***Breaks/Fragments***
**LY-R**				
Untreated (DMSO)	**213**	**0.037**^**b**^ **± 0.012**	**0.075**^**b**^ **± 0.021**	**0.056**
Treated (DNA-PKcsi)	**201**	**0.088**^**a**^ **± 0.048**	**0.279**^**a**^ **± 0.011**	**0.277**
**LY-S**				
Untreated (DMSO)	**189**	**0.159**^**b**^ **± 0.025**	**0.126**^**b**^ **± 0.003**	**0.140**
Treated (DNA-PKcsi)	**200**	**0.249**^**a**^ **± 0.046**	**0.410**^**a**^ **± 0.027**	**0.212**

Frequency of Robertsonian fusion, end-to-end telomeric fusions and DNA-DSB/fragments were recorded from two independent experiments in the LY-R and LY-S cell lines. The inhibition was induced for 24 -hour period before metaphase preparation. DMSO was used as control treatment. In each experiment at least 100 metaphases were scored (except in LY-S untreated). S.D. was used as measure of dispersion from the mean.

^a^ The difference in mean event/cell between untreated and treated cell lines are significant (p < 0.05).

^b^ The difference in mean event/cell between untreated cell lines are significant (p < 0.0001).

There was a 2.5-fold increase in the levels of spontaneous chromosome breaks in LY-S cells relative to LY-R cells (Table 1 and Figure 1). The elevated levels of endogenous chromosome breaks observed in the LY-S cell line probably reflect lack of functional DSB repair machinery in these cells as shown previously [17].

To inhibit DNA-PKcs activity we used a synthetic inhibitor IC86621. This is a highly specific inhibitor shown to generate telomere dysfunction phenotypes in mammalian cells by directly affecting DNA-PKcs activity [1,20]. Interestingly, inhibition of DNA-PKcs activity caused significant increases in frequencies of RB fusions, telomere fusions and chromosomal breaks and fragments in both cell lines (Table 1). These increases in levels of chromosome fusions in both LY-R and LY-S treated cell lines are indicative of telomere dysfunction resulting from inhibition of DNA-PKcs which is consistent with a previously published study [1]. This adds further support to the view that DNA-PKcs plays a role in protecting telomere function.

Moreover, we observed significant increases in the levels of telomeric fusions in the radiosensitive LY-S compared to its parental radio-resistance LY-R following DNA-PKcs induction (Table 1). The 1.5-fold increases in telomere fusions in the LY-S cells indicates the fragility and sensitivity of the telomeric-end protection in the LY-S cells compared to the parental radio-resistance LY-R cells, and the importance of DNA-PKcs in maintaining telomere-end function. We next asked the question whether inhibition of DNA-PKcs can affect telomere length in the telomerase-positive LY-S and LY-R mouse lymphoma cell lines.

### Telomere length shortening following inhibition of DNA-Pkcs in the LY-S and LY-R cell lines

**Figure 2:**
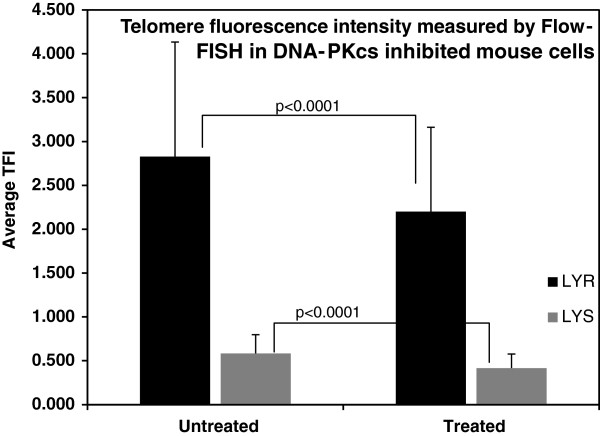
**Flow-FISH analysis of telomere length of mouse cell lines following inhibition of DNA-PKcs activity.** A reduction in TFI unit where observed when both mouse cell lines were treated with DNA-PKcs inhibitor. The difference between the percentage reduction between the treated and untreated were statistically significant (p < 0.0001). TFI unit were measured from at least 1000 cells in G0/G1 phase of cell cycle in each experiment from a total of seven independent experiments. The error bars represent s.d.

To this end we analyzed telomere length using the Flow-FISH method as described previously [21]. Our results showed a ~ 5-fold difference in telomere length between untreated LY-R and LY-S cells. Although, it has been previously shown that telomeres in LY-R cells are ~ 6.9-fold longer relative to their LY-S cell counterparts [13], the smaller difference observed here may be explained by the natural variation in telomere length in these two cell lines. For example, Cabuy *et al.* (2004) found that average telomere length in the LY-R cell line can vary between 40-60% in the same population of cells cultured for more than 20 population doublings. This natural fluctuation in telomere length may explain why we observed only a 5-fold difference (Figure 2) as opposed to the previously published 6.9-fold difference [21].

However, when the DNA-PKcs activity was inhibited this caused a reduction in the average telomere length by 27% and 21% in LY-S and LY-R cell lines respectively when compared to their untreated counterparts following a 24 hour treatment with IC86621 (Figure 2). The percentage difference in telomere fluorescence intensity between treated and untreated cell lines was statistically significant (p < 0.05) (Figure 2). This rapid significant reduction in telomere lengths in both cell lines could contribute to increases in telomeric fusions &/or increased DNA-DSBs and fragments observed above (Table 1). This further underlies the importance of DNA-PKcs in maintaining telomere function and also suggests that DNA-PKcs may be involved in telomere length regulation in mouse cells. Collectively, our data suggests that inhibition of DNA-PKcs, through exposure of cells to IC86621, causes not only loss of telomere function (Table 1, Figure 1) but also rapid telomere shortening in mouse lymphoma LY-R and LY-S cells (Figure 2). These cells show a robust telomerase activity [13] suggesting that all these changes occur independently of telomerase.

### Inhibition of DNA-PKcs causes shorter telomere length in human normal and Artemis defective primary cells

Artemis is a protein involved in repair of DNA DSBs through the NHEJ pathway and it is phosphorylated by DNA-PKcs [11,22]. It has been demonstrated that Artemis is required for repair of a subset radiation-induced DNA DSB in an ATM-dependent manner [23] and that defective Artemis patients show radiosensitivity to high [23] and low [10] doses of IR. Artemis defective patient also show immunodeficiency [24], a combined syndrome known as RS (radio sensitive)-SCID. We have demonstrated previously that cells from Artemis-defective patients exhibit mild telomere dysfunction, increased levels of irreparable telomere damage as measured by the telomere dysfunction induced foci (TIF) assay [10]. Similarly, inhibition of DNA-PKcs via IC86621 or its knock-down via RNAi in Artemis defective cells caused a slower DNA damage repair kinetics at telomeres suggesting that both proteins are needed for the stable maintenance of telomere function in human cells [10]. However, we have not analysed telomere length and frequencies of telomeric fusions in our previous study.

**Figure 3:**
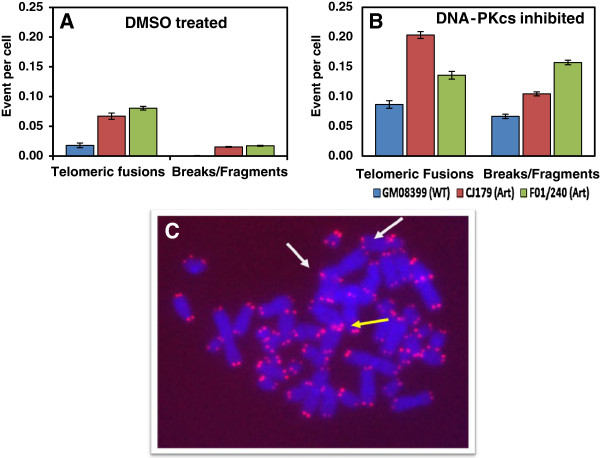
**Telo-FISH result following DNA-PKcs inhibition in human Artemis defective primary cell lines.** Two Artemis defective (CJ179, F01/240) and normal (GM08399) human primary cell lines were subjected to 200μM of DNA-PKcs inhibitor, IC86621, for twenty-four hour period. Total levels of telomeric fusions, including chromosome type, chromatid type, sister chromatic unions and chromosome ring fusions were scored in three independent experiments. Panel (**A**) is DMSO treated and panel (**B**) is DNA-PKcs inhibited. Levels of DNA-DSB chromatid fragments were also scored as breaks/fragments. Error bars indicate standard error of mean (SEM).

Here, we analysed frequencies of telomeric fusions and telomere length after DNA-PKcs inhibition, via IC86621, in two primary Artemis defective fibroblast cell lines. Inhibition of DNA-PKcs resulted in a ~50% reduction of DNA-PKcs protein activity as previously described [10]. Cytological analysis revealed a significant elevation in telomeric fusions in the two Artemis defective cell lines relative to the normal control cell line (p < 0.05) (Table 2). The majority of telomeric fusions observed were of chromatid type consistent with the observed effect of DNA-PKcs inhibition described in other studies (Bailey *et al.* 2004) (Figure 3C). Similarly, the frequency of chromosome breaks in all three cell lines was increased in the treated samples in comparison to untreated counterparts (Table 2). This is suggestive of the importance of DNA-PKcs in the efficient repair of spontaneous DNA DSB and genomic stability.

**Table 2:** Analysis of total telomeric fusions in the primary human fibroblast cells

		**Frequency (event/cell)**	
**Cell Line**	**Metaphase cells analyzed**	**Total telomeric fusions**	**Breaks/Fragments**
**GM08399 (WT)**			
Untreated (DMSO)	**111**	**0.018 ± 0.015**	**0.000 ± 0.000**
Treated (DNA-PKcsi)	**150**	**0.087 ± 0.028**	**0.067 ± 0.007**
**CJ179 (Art)**			
Untreated (DMSO)	**194**	**0.067 ± 0.026**	**0.015 ± 0.009**
Treated (DNA-PKcsi)	**182**	**0.203 ± 0.047**	**0.104 ± 0.005**
**F01-240 (Art)**			
Untreated (DMSO)	**174**	**0.081 ± 0.026**	**0.017 ± 0.012**
Treated (DNA-PKcsi)	**140**	**0.136 ± 0.021**	**0.157 ± 0.062**

Frequencies of break/fragments in two Artemis defective human primary cells and normal human primary counterpart induced with DNA-PKcs inhibitor from two independent experiments are shown. Numbers indicated are mean ± s.d.

**Figure 4:**
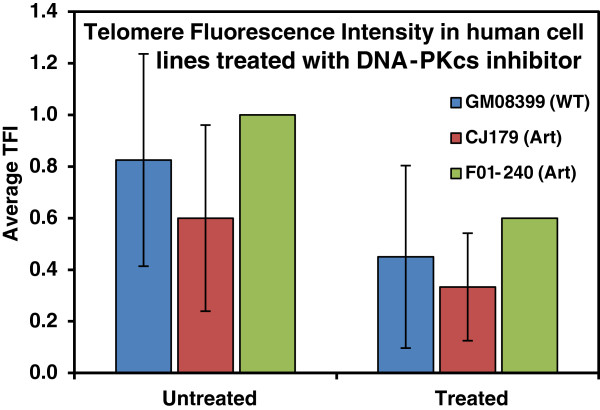
**Flow-FISH analysis of telomere lengths in human primary fibroblasts.** TFI units measured from at least four independent experiments (two for F01-240) with DMSO served as untreated control and IC86621 as inhibitor of DNA-PKcs. Error bars represent s.d. The difference in telomere length was not statistically significant.

Next, we examined the effect of DNA-PKcs inhibition on telomere length in the same cell lines. The treatment of all three cell lines with the DNA-PKcs inhibitor for a period of 24 hour caused a reduction in the in telomere length (Figure 4, Table 3). However, the difference in telomere length between untreated and treated cell lines was not significant (p < 0.164 for GM08399, p < 0.265 for CJ179) in contrast to a significant difference observed in the mouse LY-R and LY-S cells. It is therefore possible that DNA-PKcs could affect telomere length regulation mechanisms in human and mouse cell lines differently. In line with this possibility it has been suggested that the regulation of DNA-PKcs activity in human cells may be different from that in others species such as mouse [25]. However, longer incubation of the human cells with the DNA-PKcs inhibitor may produce a more significant reduction in telomere lengths in the radio-sensitive cells.

**Table 3:** Telomere length measurement in primary human cells measured by Flow-FISH

	**Telomere length in (kb)**		
	**Untreated**	**Treated**	**% change**
**GM08399 (WT)**	**5.97 ± 4.25**	**4.42 ± 4.02**	**26**
**CJ179 (Art)**	**5.00 ± 3.82**	**3.94 ± 3.60**	**21**
**F01-240 (Art)**	**6.69**	**5.04**	**25**

The TFI units (see Figure 4) were converted into kb using the standardized formula (see material and methods). The decline in telomere length in DNA-PKcs inhibited samples was observed following 24-hours of treatment. The TFI were measured from at least four independent experiments (except from one experiment for F01-240) before the mean TFI’s were converted into kb. Mean Telomere length is shown ± s.d.

The mechanism of telomere shortening in the DNA-PKcs induced immortal mouse lymphoma cells is unknown and needs to be further investigated. The future experiments should attempt to address the following questions; is telomere length shortening a progressive phenomenon in the absence of DNA-PKcs in both mouse and human cells or does it occur only for one/or two cell cycles? How does telomerase in the mouse lymphoma cells play a role in maintaining telomere length following inhibition of DNA-PKcs? The observed telomere length shortening in both human and mouse cells following a partial reduction in DNA-PKcs activity was indeed interesting and merits further investigation as to the mechanisms of DNA-PKcs activity on telomere length in radio-sensitive mouse and human cells.

## Conclusion

Our results indicate that inhibition of DNA-PKcs activity via IC86621 causes a rapid telomere shortening in both mouse lymphoma cell lines, in spite of active telomerase. Furthermore, we observed elevated frequencies of Rb and telomeric fusions in the same cell lines suggesting that DNA-PKcs inhibition affects both telomere length and function. However, the effect of DNA-PKcs in Artemis defective human fibroblasts had slightly different consequences in that telomere shortening was not significant after treatment with IC86621. This potentially reflects differences between mouse and human cells in terms of DNA-PKcs function and consequently the role that DNA-PKcs plays at telomeres.

## Materials and methods

### Cell culture conditions

Mouse lymphoma LY-R and LY-S were grown in suspension in RPMI 1640 medium (Gibco/Invitrogen) supplemented with 10% fetal calf serum (Gibco/Invitrogen) at 37°C in the atmosphere of 10% CO_2_. LY-R and LY-S cells were subcultured 1:10 ratio every two to three days, preferably before the medium colour changed to yellow.

Human Artemis defective primary fibroblast cell lines (CJ179 and F01/240) and normal human primary fibroblast cell line (GM08399) were grown in Dulbecco’s modified Eagle medium (D-MEM) supplemented with 10% fetal calf serum at 37°C with 10% CO_2_ as previously reported [10]. Cells were subcultured 1:4 ratio every three to four days depending on the confluency level.

### Inhibition of DNA-PKcs

IC86621 (Sigma Aldrich, CAS # 404009-40-1) was used at a final concentration of 200μM for 24 hours to inhibit catalytic subunit activity of DNA-PK. It has been shown previously [1,10,20] that incubation of mammalian cells with IC86621 for a period of 24hrs generates telomere dysfunction as a direct result of inhibition of DNA-PKcs activity. Cells were then used to analyze levels of telomere dysfunction and telomere length following disruption of DNA-PKcs activity.

### Telo-FISH

Telomere dysfunction analysis were performed on metaphase spread using telomere-fluorescence in situ hybridization as described previously [10]. In brief, a synthetic short oligonucleotide sequence specific to consensus telomeric sequence (CCCTAA)_3_ labelled with Cy3 PNA probe (Applied Biosystems) were hybridized to metaphase spreads at 65°C for two minutes. Prior to hybridization slides were washed in PBS, fixed with formaldehyde and digested with pepsin. After hybridization, slides were left in a humidified chamber for two hours and washed in 70% formamide. DAPI was added once slides were air dried and sealed with coverslip. All metaphase spreads were scored using Zeiss Axioplan2 microscope and images were captured using a CCD camera mounted on the microscope and captured and analyzed with MetaSystem image acquisition software (Image Associate). Over one hundred metaphase spreads were scored from each cell line for evidence of telomere fusion.

### FLOW-FISH

*In vitro* measurement of telomere length can be performed using quantitative-fluorescence *in vitro* hybridization (Q-FISH) technique on metaphase spreads or using fluorescence activated cell sorting (FACS) machine to measure fluorescently labelled telomere sequences in high-throughput. A detailed method of this technique has been described previously [21]. In brief, 5x10^5^ mouse lymphoma cells grown in suspension were collected, washed in PBS at least once. Cell pellets were then hybridized in the hybridization buffer containing 500μl of hybridization mixture of 70 percent formamide, 20mM Tris–HCl pH 7.0, 1 percent BSA made in PBS, and 0.3μg/ml of Fluorescein isothiocyanate (FITC) conjugated peptide nucleic acid (PNA) probe (CCCTAA)_3_. The hybridization mixture was heated to 80°C for ten minutes. Samples without telomeric PNA probe were used as negative control to measure background fluorescence and were normalized against the positive control samples. All samples were kept in dark for two hours to hybridize. A series of washes were done post hybridization to remove excess unbound PNA probes. Wash solutions contained 70 percent formamide, 10mM Tris–HCl, 0.1 percent BSA in PBS and 0.1 percent Tween-20. Samples were centrifuged at 3,000 rpm for five minutes after each wash. Second washes were done again twice using a 500μl solution containing PBS, 0.1 percent BSA and 0.1 percent Tween-20 and cells were centrifuged at 2000rpm for five minutes. A second incubation was done with propidium iodide (PI) (Sigma) containing PBS, 0.1 percent BSA, 10μg/ml of RNase A, and 0.1μg/ml of PI to quantitatively assess the DNA content of cells. The samples were incubated in the dark for 45 minutes to one hour at 4°C. The samples were kept on ice all the time prior to the measurement with the FACS machine. FACS Coulter EPICS XL (Beckman Coulter) was calibrated using flow-check fluorospheres (Beckman Coulter) before each measurement. The software was calibrated to measure the FITC-tagged telomeric signal on the FL1 channel, and the PI signal on FL3 channel. Cells were electronically gated for the G0/G1 phase of cell cycles form the FL3 histogram window. The Telomeric fluorescence intensity (TFI) of cells in the G0/G1 stage was recorded. TFI from the negative control cells was also measured and subtracted from the main sample reading to remove the background reading. TFI readings from a minimum of 5,000 cells and a maximum of 20,000 cells were recorded and TFI units were converted into base pairs using the formula y = 4.13x + 2.56 (R2 = 1) [13]. The accuracy of this formula was tested using LY-R and LY-S mouse cell lines.

### Statistical analysis

Student t-test was used to compare the mean of two samples with confidence interval set at 95% and α = 0.05. Standard error of mean (SEM) and Standard deviation (SD) was used as measure of dispersion.

## Abbreviations

TFI: Telomere fluorescence intensity; DNA-DBS: DNA double strand break; LY-S: Mouse lymphoma radiosensitive cells; LY-R: Mouse lymphoma radio-resistant cells; TIF: Telomere dysfunction induced foci; RB fusion: Robertsonian chromosome fusion; DNA-PKcs: Catalytic subunit of DNA protein kinase (DNA-PK); DNA-PKcsi: Inhibited DNA-PKcs using IC86621; RS-SCID: Radiosensitive-severe combined immune-deficiency

## Competing interest

Authors declare no competing interests.

## Authors’ contribution

HY and PS designed experiments. HY performed all experiments. HY and PS wrote the manuscript. All authors read and approved the final manuscript.

## References

[B1] Bailey SM Brenneman MA Halbrook J Nickoloff JA Ullrich RL Goodwin EH The kinase activity of DNA-PK is required to protect mammalian telomeres DNA Repair (Amst) 2004 3 3 225 233 10.1016/j.dnarep.2003.10.013 15177038

[B2] Frias C Pampalona J Genesca A Tusell L Telomere dysfunction and genome instability Front Biosci 2012 17 2181 2196 10.2741/4044 22652771

[B3] de Lange T Protection of mammalian telomeres Oncogene 2002 21 4 532 540 10.1038/sj.onc.1205080 11850778

[B4] Goytisolo FA Blasco MA Many ways to telomere dysfunction: in vivo studies using mouse models Oncogene 2002 21 4 584 591 10.1038/sj.onc.1205085 11850783

[B5] Palm W de Lange T How shelterin protects mammalian telomeres Annu Rev Genet 2008 42 301 334 10.1146/annurev.genet.41.110306.130350 18680434

[B6] Slijepcevic P DNA damage response, telomere maintenance and ageing in light of the integrative model Mech Ageing Dev 2008 129 1–2 11 16 18063009 10.1016/j.mad.2007.10.012

[B7] Bailey SM Meyne J Chen DJ Kurimasa A Li GC Lehnert BE Goodwin EH DNA double-strand break repair proteins are required to cap the ends of mammalian chromosomes Proc Natl Acad Sci U S A 1999 96 26 14899 14904 10.1073/pnas.96.26.14899 10611310 PMC24745

[B8] Espejel S Franco S Sgura A Gae D Bailey SM Taccioli GE Blasco MA Functional interaction between DNA-PKcs and telomerase in telomere length maintenance EMBO J 2002 21 22 6275 6287 10.1093/emboj/cdf593 12426399 PMC137185

[B9] Slijepcevic P Al-Wahiby S Telomere biology: integrating chromosomal end protection with DNA damage response Chromosoma 2005 114 4 275 285 10.1007/s00412-005-0338-4 15843951

[B10] Yasaei H Slijepcevic P Defective Artemis causes mild telomere dysfunction Genome Integr 2010 1 1 3 10.1186/2041-9414-1-3 20678254 PMC2907561

[B11] Ma Y Pannicke U Lu H Niewolik D Schwarz K Lieber MR The DNA-dependent protein kinase catalytic subunit phosphorylation sites in human Artemis J Biol Chem 2005 280 40 33839 33846 10.1074/jbc.M507113200 16093244

[B12] Peddi P Loftin CW Dickey JS Hair JM Burns KJ Aziz K Francisco DC Panayiotidis MI Sedelnikova OA Bonner WM DNA-PKcs deficiency leads to persistence of oxidatively induced clustered DNA lesions in human tumor cells Free Radic Biol Med 2010 48 10 1435 1443 10.1016/j.freeradbiomed.2010.02.033 20193758 PMC2901171

[B13] McIlrath J Bouffler SD Samper E Cuthbert A Wojcik A Szumiel I Bryant PE Riches AC Thompson A Blasco MA Telomere length abnormalities in mammalian radiosensitive cells Cancer Res 2001 61 3 912 915 11221881

[B14] Wong HP Slijepcevic P Telomere length measurement in mouse chromosomes by a modified Q-FISH method Cytogenet Genome Res 2004 105 2–4 464 470 15237235 10.1159/000078220

[B15] Szumiel I L5178Y sublines: a look back from 40 years. Part 2: response to ionizing radiation Int J Radiat Biol 2005 81 5 353 365 10.1080/09553000500143534 16076750

[B16] Sprung CN Davey DS Goh SK Radford IR McKay MJ Uncoupling of telomere length and radiosensitivity in mouse lymphoma cell lines of similar genetic background Int J Radiat Biol 2007 83 8 515 521 10.1080/09553000701452270 17613124

[B17] Wlodek D Hittelman WN The repair of double-strand DNA breaks correlates with radiosensitivity of L5178Y-S and L5178Y-R cells Radiat Res 1987 112 1 146 155 10.2307/3577085 3659295

[B18] Jaworska A Szumiel I De Angelis P Olsen G Reitan J Evaluation of ionizing radiation sensitivity markers in a panel of lymphoid cell lines Int J Radiat Biol 2001 77 3 269 280 10.1080/09553000010019638 11258841

[B19] Slijepcevic P Telomeres and mechanisms of Robertsonian fusion Chromosoma 1998 107 2 136 140 10.1007/s004120050289 9601982

[B20] Kashishian A Douangpanya H Clark D Schlachter ST Eary CT Schiro JG Huang H Burgess LE Kesicki EA Halbrook J DNA-dependent protein kinase inhibitors as drug candidates for the treatment of cancer Mol Cancer Ther 2003 2 12 1257 1264 14707266

[B21] Cabuy E Newton C Roberts T Newbold R Slijepcevic P Identification of subpopulations of cells with differing telomere lengths in mouse and human cell lines by flow FISH Cytometry A 2004 62 2 150 161 15523603 10.1002/cyto.a.20096

[B22] Ma Y Pannicke U Schwarz K Lieber MR Hairpin opening and overhang processing by an Artemis/DNA-dependent protein kinase complex in nonhomologous end joining and V(D)J recombination Cell 2002 108 6 781 794 10.1016/S0092-8674(02)00671-2 11955432

[B23] Riballo E Kühne M Rief N Doherty A Smith GC Recio MJ Reis C Dahm K Fricke A Krempler A A pathway of double-strand break rejoining dependent upon ATM, Artemis, and proteins locating to gamma-H2AX foci Mol Cell 2004 16 5 715 724 10.1016/j.molcel.2004.10.029 15574327

[B24] Noordzij JG Verkaik NS van der Burg M van Veelen LR de Bruin-Versteeg S Wiegant W Vossen JM Weemaes CM de Groot R Zdzienicka MZ Radiosensitive SCID patients with Artemis gene mutations show a complete B-cell differentiation arrest at the pre-B-cell receptor checkpoint in bone marrow Blood 2003 101 4 1446 1452 10.1182/blood-2002-01-0187 12406895

[B25] van der Burg M van Dongen JJ van Gent DC DNA-PKcs deficiency in human: long predicted, finally found Curr Opin Allergy Clin Immunol 2009 9 6 503 509 10.1097/ACI.0b013e3283327e41 19823081

